# Leptin Contributes to the Adaptive Responses of Mice to High-Fat Diet Intake through Suppressing the Lipogenic Pathway

**DOI:** 10.1371/journal.pone.0006884

**Published:** 2009-09-03

**Authors:** Lei Jiang, Qiong Wang, Yue Yu, Feng Zhao, Ping Huang, Rong Zeng, Robert Z. Qi, Wenjun Li, Yong Liu

**Affiliations:** 1 Key Laboratory of Nutrition and Metabolism, Institute for Nutritional Sciences, Shanghai Institutes for Biological Sciences, Chinese Academy of Sciences, Shanghai, China; 2 Key Laboratory of Systems Biology, Shanghai Institutes for Biological Sciences, Chinese Academy of Sciences, Shanghai, China; 3 Department of Biochemistry, Hong Kong University of Science and Technology, Clear Water Bay, Kowloon, Hong Kong, China; Sapienza University of Rome, Italy

## Abstract

**Background:**

Leptin is an adipocyte-derived hormone that plays a critical role in energy homeostasis and lipid metabolism. Overnutrition-associated obesity is known to be accompanied by hyperleptinemia. However, the physiological actions of leptin in the metabolic responses to high-fat diet (HFD) intake remain to be completely elucidated. Here we characterized the metabolic features of mice fed high-fat diets and investigated the impact of leptin upon the lipogenic program which was found to be suppressed by HFD feeding through a proteomics approach.

**Results:**

When maintained on two types of high-fat diets for up to 16 weeks, mice with a higher fat intake exhibited increased body fat accumulation at a greater pace, developing more severely impaired glucose tolerance. Notably, HFD feeding at 4 weeks elicited the onset of marked hyperleptinemia, prior to the occurrence of apparent insulin resistance and hyperinsulinemia. Proteomic analysis revealed dramatically decreased expression of lipogenic enzymes in the white adipose tissue (WAT) from HFD-fed mice, including ATP-citrate lyase (ACL) and fatty acid synthase (FAS). The expression of ACL and FAS in the liver was similarly suppressed in response to HFD feeding. By contrast, HFD-induced downregulation of hepatic ACL and FAS was significantly attenuated in leptin receptor-deficient *db/db* mice. Furthermore, in the liver and WAT of wild type animals, intraperitoneal leptin administration was able to directly suppress the expression of these two lipogenic enzymes, accompanied by reduced triglyceride levels both in the liver and serum.

**Conclusions:**

These results suggest that leptin contributes to the metabolic responses in adaptation to overnutrition through suppressing the expression of lipogenic enzymes, and that the lipogenic pathway represents a key targeted peripheral component in exerting leptin's liporegulatory actions.

## Introduction

As a major risk factor for type 2 diabetes and cardiovascular complications, obesity is currently reaching epidemic proportions worldwide [1], largely stemming from complex interactions between genetic factors and environmental influences such as overnutrition. In mammals, multiple mechanisms act in an integrated manner to balance energy storage and expenditure, and chronic disruption of energy balance leads to excessive accumulation of fat in the adipose tissue [2]. In addition to energy storage, the adipose tissue is also known to serve as a critical endocrine organ that releases a variety of adipokines, eliciting an array of metabolic effects on lipid and glucose metabolism [3]. Leptin is an adipocyte-secreted hormone that plays a critical role in energy homeostasis [4]–[6]. Primarily acting through activation of leptin receptor-expressing neurons in the hypothalamus [7]–[11], leptin functions to control energy balance and the fat mass via reducing food intake and increasing energy expenditure. On the other hand, leptin can also exert crucial metabolic effects upon lipid metabolism, preventing triglyceride (TG) accumulation in peripheral tissues [12]. For instance, it has been shown that leptin is able to stimulate fatty acid oxidation through activation of AMP-activated protein kinase (AMPK), subsequently inhibiting acetyl-CoA carboxylase (ACC) activity, in the skeletal muscle [13]. Increased circulating levels of leptin (i.e. hyperleptinemia) have been found to be associated with obesity induced by overnutrition, as in the case of chronic intake of high-fat diet [14]. However, whether hyperleptinemia exerts its metabolic liporegulatory actions and represents an adaptive response to chronic overnutrition has yet to be completely understood.

*De novo* lipogenesis in the liver and WAT plays a key role in body's energy storage and is coordinately controlled in response to nutritional, hormonal and metabolic stimuli [15], [16]. This cytosolic process occurs with the initial conversion of citrate to acetyl-CoA catalyzed by ATP-citrate lyase (ACL) [17], [18]. Acetyl-CoA is further converted to malonyl-CoA by ACC, the rate-limiting step in *de novo* fatty acid synthesis [19]. Malonyl-CoA is then used as the substrate of fatty acid synthase (FAS) for fatty acid synthesis [20]. Recently, adipose tissue lipogenesis has been shown to be controlled by leptin via STAT3-independent central mechanisms [21]; whereas a liporegulatory role of hyperleptinemia has been implicated in non-adipose tissues, affecting lipogenesis and fatty acid oxidation [22]. Therefore, it is likely that leptin may act upon the peripheral lipogenic program in the face of overnutrition to mediate body's metabolic adaptation responses.

Using the well-established HFD-induced obesity mouse model, we employed a proteomic approach to examine the global protein expression changes in the WAT as associated with the progression of adiposity. We found that, among protein enzymes involved in lipid metabolism, the lipogenic enzymes ACL and FAS were predominantly suppressed in both the WAT and liver in mice challenged by HFD feeding, in parallel with concomitant onset of hyperleptinemia. Then we further investigated the impact of leptin upon the control of lipogenic enzyme expression in the liver and WAT.

## Results

### High-fat diet feeding induces hyperleptinemia prior to hyperinsulinemia and insulin resistance

**Figure 1:**
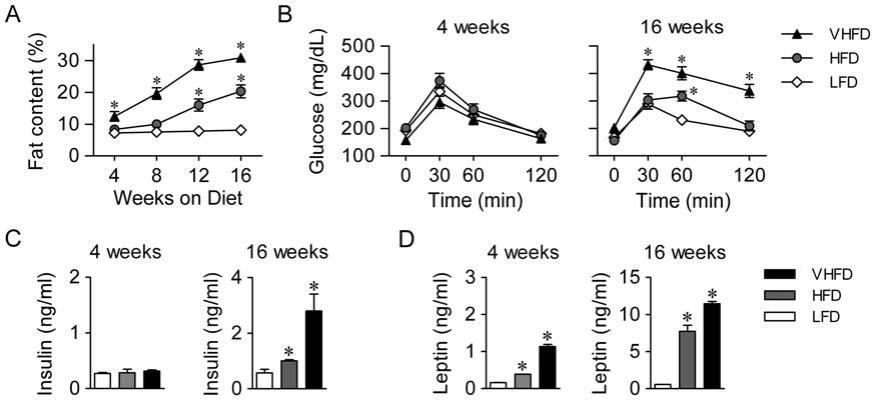
High-fat diet feeding elicits hyperleptinemia prior to hyperinsulimia. C57BL/6 male mice at 6 weeks of age were fed LFD, HFD and VHFD, respectively. (A) Body fat accumulation was monitored monthly by nuclear magnetic resonance (NMR) and shown as percentage of total body weight (n = 15/group). (B) Glucose tolerance tests were performed in mice at 4 weeks (n = 10/group) or 16 weeks (n = 15/group) as indicated. (C, D) Serum concentrations of (C) insulin and (D) leptin were determined in mice maintained on the diets for 4 or 16 weeks (n = 3–4/group). All data are presented as mean±SEM. ^*^p<0.05 vs. LFD-fed mice.

To characterize the metabolic features that accompany the progression of adiposity, male C57BL/6 mice were fed low (LFD, 10% fat), high (HFD, 45% fat) and very high (VHFD, 60% fat) fat diets, respectively. In comparison with control mice fed LFD, mice fed HFD or VHFD developed overt obesity at 16 weeks, with their body fat content increased by ∼2.5-fold and ∼3.8-fold, respectively (**Figure 1A**). Whereas HFD feeding for 4 weeks did not show significant effects, VHFD feeding considerably increased adiposity (by 1.7-fold as compared to LFD), indicating that intake of higher dietary fat (60% fat) induces a higher degree of obesity at a faster pace. Moreover, supporting the close correlation between insulin resistance and obesity, mice fed VHFD exhibited more pronounced glucose intolerance at 16 weeks but not at 4 weeks when compared with LFD-fed animals (**Figure 1B**). Consistently, marked hyperinsulinemia was found at 16 weeks in HFD-fed mice, but not at 4 weeks (**Figure 1C**). In contrast, despite modest increases in body fat mass (by ∼70%) in VHFD-fed mice at 4 weeks, a ∼7-fold upsurge in circulating leptin levels was observed relative to LFD-fed mice; whereas at 16 weeks both HFD and VHFD feeding led to prominent degrees of hyperleptinemia (**Figure 1D**). These data demonstrate that the onset of hyperleptinemia occurs prior to that of hyperinsulinemia and insulin resistance during the progressive development of diet-induced obesity, which may exert metabolic actions in response to HFD feeding.

### Lipogenic enzymes are predominantly suppressed in response to HFD feeding

**Figure 2:**
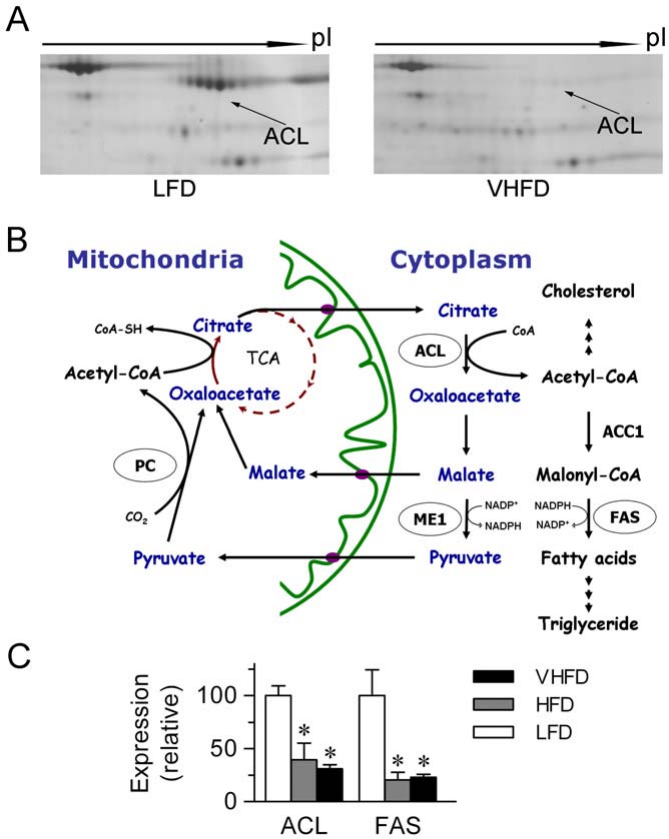
Identification by proteomic analysis of lipogenic enzymes suppressed in the white adipose tissue in response to the high-fat diet challenge. (A) Protein extracts of the epididymal fat pad from mice fed LFD vs. VHFD for 4 weeks (n = 3/group) were fractionated by 2D SDS-PAGE. Proteins of interest were subsequently subjected to tryptic peptide identification analysis using LC-mass spectrometry. Shown is a representative section of the silver-stained 10% 2D SDS-PAGE gel with the migration of ACL protein indicated by the arrows. (B) Schematic diagram of the sequential steps in *de novo* lipogenesis as related to the TCA cycle in the mitochondrion. Highlighted by circles are the identified enzymes displaying predominantly decreased expression levels. (C) Real-time quantitative RT-PCR analysis of the mRNA expression levels of ACL and FAS in the WAT of mice fed LFD vs. VHFD for 4 weeks, shown as mean±SEM (n = 3/group).^ *^p<0.05 vs. LFD-fed mice.

To explore the global protein expression features in the WAT of mice that may manifest the metabolic impact of HFD feeding, we took a proteomic approach to analyze the adipose protein extracts from mice fed LFD versus VHFD by quantitative two-dimensional (2D) polyacrylamide gel electrophoresis (PAGE) (**Figure 2A**). Subsequent tryptic peptide analysis by LC-mass spectrometry identified a total of 33 proteins in mice fed VHFD for 4 weeks and of 22 proteins in mice fed VHFD for 16 weeks, respectively, that exhibited significantly altered expression levels (**Table 1** **and Supporting Information Table S1**). Included among the proteins upregulated are several stress response- or detoxification-related proteins (such as heat shock cognate 71 kDa protein) and cytoskeleton/structural proteins, whereas most of the proteins showing decreased expression levels were involved in lipid, fatty acid and carbohydrate metabolism. Notably, lipogenesis-related enzymes, including ACL, FAS, transketolase and malic enzyme 1 (ME1), displayed the most prominent suppression in response to VHFD feeding. These enzymes catalyze critical steps in *de novo* fatty acid synthesis, either responsible for production of cytosolic acetyl-CoA, catalysis of fatty acid synthesis from malonyl-CoA, or generation of NADPH to be consumed in fatty acid synthesis (**Figure 2B**). Of interesting note, as analyzed by 2D-PAGE, ACL protein migrated as differentially charged species, likely arising from multiple modifications (e.g. phosphorylation). To verify the downregulated expression of lipogenic enzymes as detected by the proteomic analysis, quantitative RT-PCR assessment was performed. The mRNA expression levels of both ACL and FAS were similarly reduced by ∼80% in mice fed HFD or VHFD for 4 weeks (**Figure 2C**).

**Table 1:** Proteins with altered expression in the WAT of the HFD-fed mice at 4 weeks.

Accession No.	Gene name	Description	Fold
NP_598798	ATP citrate lyase	lipid, fatty acid and isoprenoid metabolism	−10.68
NP_032014	Fatty acid synthase	lipid, fatty acid and isoprenoid metabolism	−4.83
NP_033414	Transketolase	pentose-phosphate pathway	−2.93
NP_032641	NADP-dependent malic enzyme	lipid, fatty acid and isoprenoid metabolism	−2.78
NP_034079	Carnitine O-palmitoyltransferase II	lipid, fatty acid and isoprenoid metabolism	−2.65
NP_033788	Aldose reductase	C-compound and carbohydrate metabolism	−2.31
AAH94462	Aconitate hydratase, mitochondrial precursor	C-compound and carbohydrate metabolism	−2.13
NP_031407	Long-chain specific acyl-CoA dehydrogenase	lipid, fatty acid and isoprenoid metabolism	−2.04
NP_035164	Peroxiredoxin-1	stress response,detoxification	−1.90
AAH16619	Pyruvate kinase 3	C-compound and carbohydrate metabolism	−1.88
AAF67667	Isovaleryl-CoA dehydrogenase	amino acid metabolism	−1.79
NP_031409	Short-chain specific acyl-CoA dehydrogenase	lipid, fatty acid and isoprenoid metabolism	−1.77
NP_032823	Pyruvate carboxylase	lipid, fatty acid and isoprenoid metabolism	−1.69
AAH39925	Bifunctional purine biosynthesis protein PURH	nucleotide/nucleoside/nucleobase metabolism	−1.65
NP_080455	Abhydrolase domain-containing protein 5	protein modification	−1.55
NP_083060	Coagulation factor XIII A chain precursor	protein modification	1.65
NP_033375	Indolethylamine N-methyltransferase	C-compound and carbohydrate metabolism	1.68
NP_666232	Gelsolin	cytoskeleton/structural proteins	1.69
NP_694708	EH-domain containing 2	cytoskeleton/structural proteins	1.71
NP_036167	Synaptic vesicle membrane protein VAT-1 homolog	intracellular transport vesicles	1.72
NP_038534	Eukaryotic initiation factor 4A-II	DNA/RNA processing	1.76
AAH66191	Heat shock cognate 71 kDa protein	stress response,detoxification	1.80
NP_067248	Creatine kinase B-type	amino acid metabolism	1.81
AAH55341	Rab GDP dissociation inhibitor beta-2	cytoskeleton/structural proteins	1.94
NP_075608	Alpha-enolase	C-compound and carbohydrate metabolism	2.06
AAC53295	Proteasome activator complex subunit 1	protein/peptide degradation	2.29
NP_031978	Protein disulfide isomerase associated 3	signal transduction	2.35
AAA69475	Peroxiredoxin-2	stress response,detoxification	2.37
CAA31455	Gamma actin-like protein	cytoskeleton/structural proteins	2.54
NP_058662	D-3-phosphoglycerate dehydrogenase	amino acid metabolism	+
AAI08387	Actin-like protein 3	cytoskeleton/structural proteins	+
NP_033768	Cysteine-rich secretory protein 1 precursor	extracellular matrix component	+
NP_033861	Aldose reductase-related protein 1	lipid, fatty acid and isoprenoid metabolism	+

Numbers with “−” indicate fold decreases in the VHFD-fed group, or fold increases otherwise. “+” indicates that the protein was only detectable in the WAT from the VHFD-fed mice. Quantitation was done using the ImageMaster™ 2D Elite Software (Amersham Biosciences).

**Figure 3:**
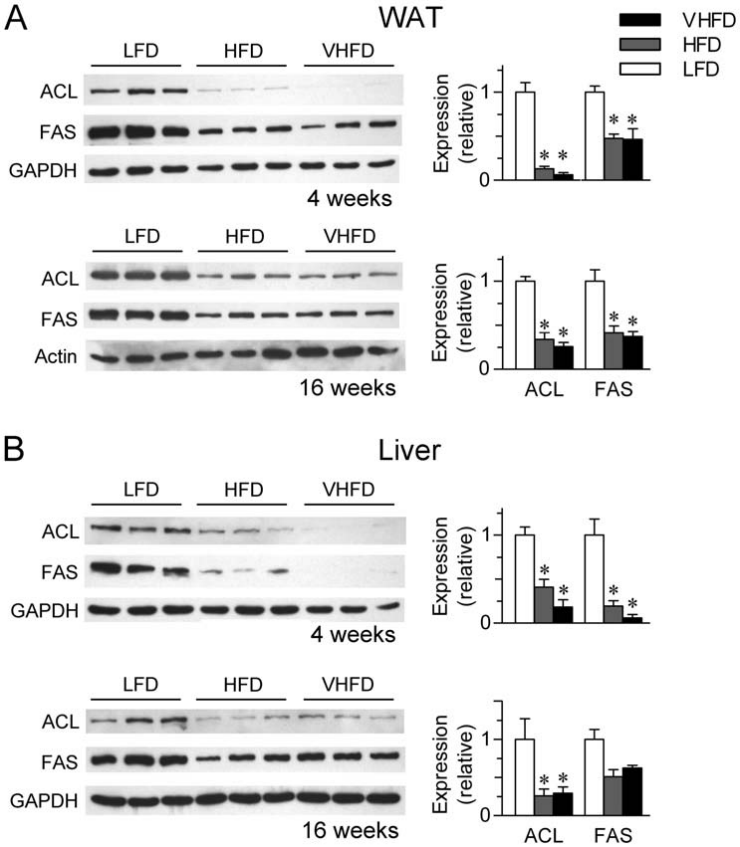
High-fat diet feeding suppresses the expression of lipogenic enzymes both in the white adipose tissue and liver. Western immunoblot analysis of the expression of ACL and FAS in the (A) WAT or (B) liver from mice fed LFD, HFD and VHFD for 4 and 16 weeks, respectively. Each lane represents the tissue extract from one animal. Bar graphs indicate the relative protein expression levels determined by densitometric quantification of the immunoblots after normalization to actin or GAPDH. Data are shown as mean±SEM (n = 3/group). *p<0.05 vs. LFD-fed mice.

Then we further examined by immunoblotting the HFD-induced suppression of ACL and FAS proteins in the WAT, both of which were markedly reduced at 4 or 16 weeks in HFD- or VHFD-fed mice (**Figure 3A**), consistent with their downregulated mRNA expression levels. Similarly in the liver, the other major lipogenic organ responsible for fuel conversion and storage, HFD feeding also led to dramatically suppressed expression of ACL and FAS proteins (**Figure 3B**).

Collectively, these data demonstrate that in response to high dietary fat intake, hyperleptinemia occurs at an early stage during the progressive accumulation of fat mass, concurrent with marked suppression of the lipogenic program in the adipose tissues as well as in the liver. This indicates that leptin may contribute to eliciting these metabolic changes in an adaptive response to overnutrition.

### Leptin signaling deficiency attenuates HFD-induced suppression of hepatic lipogenic enzymes

**Figure 4:**
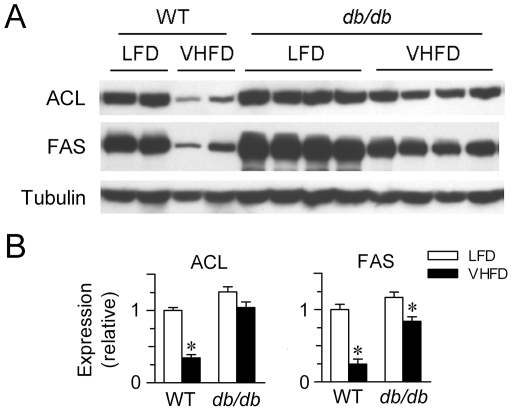
Attenuation of high-fat diet-induced suppression of hepatic lipogenic enzymes in *db/db* mice. (A) Western immunoblot analysis of ACL and FAS protein levels in the liver of WT and *db/db* mice fed LFD vs. VHFD for 4 weeks. (B) Quantification of the relative protein levels in (A) after normalization to tubulin as the loading control, shown as mean±SEM (n = 2–4/group). ^*^p<0.05 vs. LFD-fed mice of the same genotype.

To investigate the link between the metabolic effects of hyperleptinemia and the suppression of lipogenic enzymes upon the challenge of HFD, we examined the impact of HFD feeding in leptin receptor-deficient, hyperphagic and obese *db/db* mice. When fed VHFD versus LFD for 4 weeks, *db/db* mice displayed body weight gain and fat mass accumulation to a greater extent than their WT littermates (data not shown). Consistently, hepatic expression of ACL and FAS was markedly reduced in VHFD-fed WT mice; in contrast, suppression of hepatic ACL and FAS expression was prominently blunted in VHFD-fed *db/db* mice (**Figure 4**). Thus, these results suggest that fully functional leptin actions are required for HFD-induced suppression of the lipogenic enzymes in the liver, further supporting the involvement of leptin in adaptive responses to overnutrition through its suppressing action on the lipogenic program.

### Leptin administration directly suppresses the expression of lipogenic enzymes

**Figure 5:**
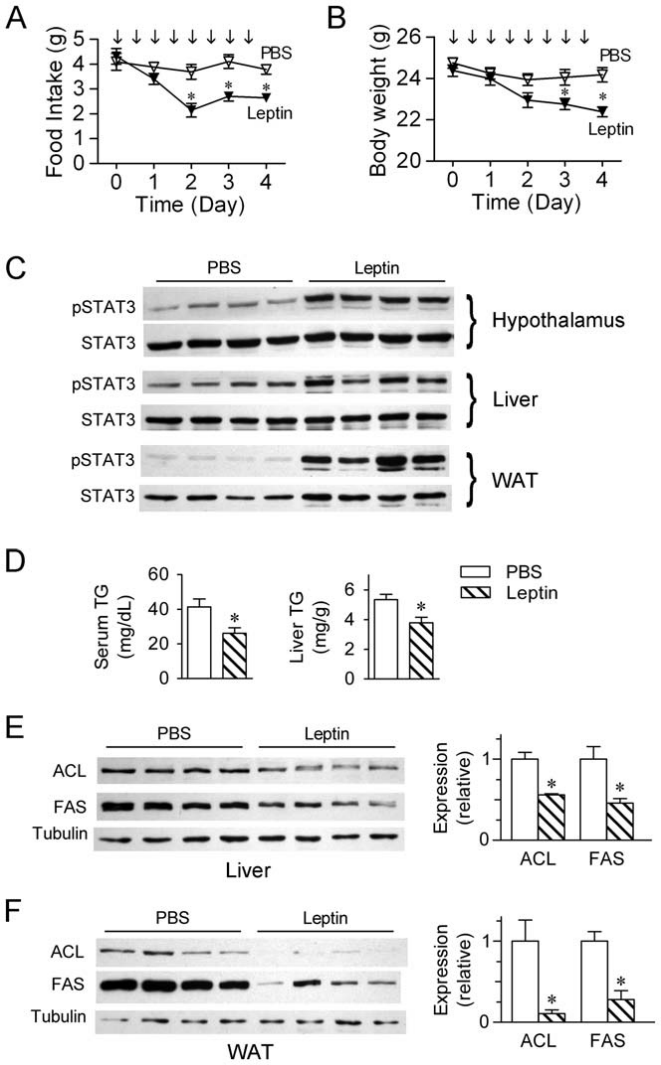
Exogenous leptin suppresses the expression of lipogenic enzymes both in the liver and white adipose tissue. C57BL/6 male mice at 8 weeks of age were treated with PBS vs. leptin (2 mg/kg) via intraperitoneal (i.p.) injection twice a day for 4 days, and were sacrificed 5 hours after the last injection. (A) Food intake and (B) body weight were measured daily before the dark cycle, and arrows indicate the time points of leptin injections. (C) Western immunoblots showing leptin-stimulated activation of STAT3 in the hypothalamus, liver and white adipose tissue, as detected by the indicated antibodies. (D) The triglyceride (TG) levels both in the serum and liver were measured after leptin administration. (E–F) Protein levels of ACL and FAS in the liver (E) and WAT (F) were analyzed by Western immunoblot, with quantification results shown in the bar graphs. Data in all panels are presented as mean±SEM (n = 4–5/group); ^*^p<0.05 vs. PBS-treatment group.

Leptin has been reported to reduce triglyceride content in peripheral tissues, including liver, muscle and pancreas [22]. To determine whether leptin exerts its liporegulatory effects through, at least in part, direct downregulation of lipogenic enzymes, we injected wild type mice maintained on a normal chow diet intraperitoneally (i.p.) with leptin twice a day for 4 days. Consistent with previously documented studies [23], leptin administration resulted in considerable reductions in food intake and body weight (**Figure 5A, B**). At 5 hours post the last injection with leptin, marked phosphorylation/activation of signal transducer and activator of transcription 3 (STAT3) were detected in the hypothalamus as well as in the WAT and liver (**Figure 5C**). In parallel, leptin treatment significantly decreased the triglyceride (TG) levels both in the serum and liver (**Figure 5D**). Immunoblot analysis showed that leptin significantly reduced the protein expression levels of ACL (by ∼46%) and FAS (by ∼56%) in the liver (**Figure 5E**); more dramatic suppression of ACL and FAS protein expression was also observed in the WAT (**Figure 5F**). Together, these data further indicate that peripheral lipogenic pathway represents a key targeted component in exerting leptin's liporegulatory actions.

## Discussion

As major organs for energy conversion and storage, both the liver and white adipose tissue play crucial roles in metabolic homeostasis by responding to body's nutritional and energy states. In this study, we show that in an adaptive response to overnutrition (HFD intake), the early onset of hyperleptinemia most likely contributes to the suppression of the lipogenic program in the liver and white adipose tissue; leptin exerts crucial liporegulatory functions via its metabolic control of the peripheral lipogenic pathway.

Taking a proteomic approach, we attempted to characterize the global protein expression profiles in the WAT of mice when challenged with HFD feeding, leading to the identification of lipogenic enzymes, including ACL and FAS, which exhibited most predominantly decreased expression patterns. Despite the limited number of proteins identified by the proteomic strategy that allowed only for identification of those expressed at abundant levels, our results are in line with the reported oligonucleotide microarray profiling studies by Moraes RC *et al.* [24]. Similar to the microarray results showing differentially expressed genes in the WAT of HFD-fed mice after 8 weeks [24], many of the WAT proteins identified were also found to be down-regulated in HFD-fed mice, among which included enzymes involved in lipid metabolism (e.g. FAS and transketolase) and detoxification processes, as well as cytoskeleton structural components. Therefore, our proteomic analysis of the WAT revealed similarly broad changes in the gene expression spectrum in response to HFD intake. Moreover, the observed suppression in the liver of the protein expression of the two key lipogenic enzymes, ACL and FAS, is also consistent with their reduced mRNA expression levels documented by hepatic cDNA microarray analyses of HFD-fed obese mice [25], [26]. As HFD-induced changes in the WAT expression of stress response proteins and cytoskeleton components may reflect the coordinate response to meet the demand for increased energy storage in adipocytes, our findings are in accordance with the notion that transcriptional adaptation occurs upon the challenge by high dietary fat intake. The observed suppression of the endogenous lipogenic program in the adipose tissue and liver most likely represents an important feature of the early adaptive responses to the state of overnutrition, whereby increased leptin levels may exert important regulatory actions.

Multiple mechanisms are thought to mediate the regulatory actions of leptin on lipid metabolism. Direct peripheral actions by leptin have been implicated in depleting fat content through increased fatty acid oxidation as well as suppressed lipogenesis [12], [22], [27]. Upon HFD feeding, we observed dramatically increased leptin but normal insulin levels that paralleled the concurrent suppression of lipogenic enzymes in the liver and WAT; on the other hand, abrogation of leptin signaling in *db/db* mice abolished, at least partially, the suppressing effects of HFD feeding on hepatic ACL and FAS expression. These results suggest that HFD-induced hyperleptinemia contributes to the suppression of the lipogenic program, consistent with previously reported findings that indicate the requirement of functional leptin actions for HFD-induced suppression of *de novo* lipogenesis using the leptin receptor-defective Zucker diabetic fatty (ZDF) rats [28], [29]. Moreover, we observed that direct leptin administration stimulated the phosphorylation activation of STAT3 not only in the hypothalamus, but also in the liver and WAT, paralleled by reduced ACL and FAS protein expression both in the liver and WAT. Whereas a central action by leptin has been shown to be critical to its metabolic control of lipid metabolism [21], [30], it remains to be completely clarified whether leptin exerts its suppressing effects upon the lipogenic program also through autonomous peripheral mechanisms, or whether the peripheral STAT3 activation is essentially involved in mediating leptin's liporegulatory actions. In addition, leptin has been demonstrated to result in more pronounced suppression of the expression of lipogenic genes, including ACL and FAS, as compared with pair-feeding in leptin-deficient *ob/ob* mice via hepatic mciroarray analysis [31], while another study by RT-PCR assessment showed similarly repressed expression of lipogenic genes, such as ACC and FAS, upon leptin treatment relative to pair-feeding in *ob/ob* mice [32]. Thus, it remains to be completely deciphered whether leptin exerts its action on the control of lipogenic program in a manner totally independent of its regulation of food intake.

Obesity is pathogenically associated with the occurrence of leptin resistance in human as well as in animal models [33], [34]. Because our results support that leptin exerts its crucial metabolic effects through controlling the expression of lipogenic enzymes, it is conceivable that dysregulated lipogenic pathway may underlie the deleterious effects of defective leptin signaling upon lipid metabolism. Indeed, as we have previously demonstrated in *db/db* mice, hepatic ACL is dysregulated in the absence of functional leptin signaling; furthermore, hepatic ACL suppression leads to marked protection of the obese mice against the development of liver steatosis [35]. Thus, our results provide physiological evidence that the lipogenic pathway serves as a key component in mediating leptin's regulatory actions in lipid homeostasis.

## Materials and Methods

### Animal studies

C57BL/6 male mice (Shanghai Laboratory Animal Co. Ltd) and C57BL/6 *db/db* male mice (from Model Animal Research Center) were housed in laboratory cages at a temperature of 23±3°C and a humidity of 35±5% under a 12-hr dark/light cycle (lights on at 6:30 am) in accredited animal facilities at Shanghai Institutes for Biological Sciences, CAS. For DIO mouse model, mice were randomly grouped with free access to one of the three experimental diets (n = 15/group) containing 10 kcal% (LFD), 45 kcal% (HFD) and 60 kcal% (VHFD) fat, respectively (Research Diets). Total body fat content was measured by nuclear magnetic resonance (NMR) using the Minispec Mq7.5 (Bruker). For leptin administration, individually caged 8-week-old male mice (n = 4–5 per group) on chow diet were first acclimated by i.p. injection of PBS for 5 days, followed by i.p. injection twice daily (6:00 pm and 8:00 am) with PBS or recombinant mouse leptin (National Hormone and Peptide Program) at a dose of 2 mg/kg for 4 days. Mice were sacrificed 5 hours after the eighth injection (at 1:00 pm). Tissues of interest were snap-frozen in liquid nitrogen immediately after resection and stored at −80°C. All experimental procedures and protocols were approved by the Institutional Animal Care and Use Committee of the Institute for Nutritional Sciences, CAS.

### 2D SDS-PAGE and protein identification by MS

Proteins extracts (30 µg) from epididymal fat pads were loaded onto an Immobiline Drystrip IPG gel (Amersham Biosciences, pH range 3–10 NL), and proteins were separated using the IPGphor Isoelectric Focusing System (Amersham Biosciences) according to manufacturer's instructions. For the second dimension analysis, the IPG strips were first equilibrated in a buffer containing 50 mM Tris-HCl, pH 8.8, 6 M urea, 30% glycerol, 2% SDS, 0.002% bromophenol blue and 1% dithiothreitol, followed by incubation in the same buffer with 1% DTT replaced by 2.5% iodoacetamide. Proteins were fractionated by 10% SDS-PAGE and visualized by silver staining, followed by quantitative analysis using the ImageMaster™ 2D Elite Software (Amersham Biosciences). Visualized protein spots of interest were then excised from Coomassie-blue-stained gels and subjected to in-gel digestion by trypsin. Recovered peptides for each protein were analyzed by nanoelectrospray tandem mass spectrometry using a quadrupole/time-of-flight (Q-TOF) hybrid mass spectrometer (QSTAR-Pulsar, Applied Biosystems/Sciex and Bruker-Daltonics AuoFlex TOF-TOF LIFT). The mass profiles of tryptic peptides were subsequently analyzed via searching protein sequence databases (NCBI Nonredundant Protein Database).

### Glucose tolerance test (GTT)

After an overnight fast, mice were injected i.p. with glucose at 1 g/kg. Blood was collected from the tail vein at 0, 30, 60, and 120 min after glucose injection, and glucose concentrations were measured by a glucometer (FreeStyle).

### Blood measurements

Serum level of triglycerides was determined by the Serum Triglyceride Determination Kit (Sigma). Serum concentrations of insulin, leptin were measured by the Mouse Insulin RIA Kit and Mouse Adipocyte LINCOplex Kit (LINCO Research), respectively, according to manufacturer's instructions.

### Antibodies and Western immunoblot analysis

Monoclonal FAS antibody was purchased from BD Biosciences; ACL, STAT3 and phospho-STAT3 antibodies were from Cell Signaling; GAPDH antibody was from KANGCHEN; Monoclonal antibody against tubulin was from Sigma. For Western immunoblotting, tissue extracts were prepared by lysis with CelLytic™ MT (Sigma) and centrifuged for 20 min at 20,000 g to remove the debris. Proteins (20∼40 µg) were separated by SDS-PAGE and transferred to PVDF filter membrane (Amersham Biosciences). After incubation with the desired antibodies, the blots were developed using Amersham's ECL-plus Detection System.

### Quantitative RT-PCR analysis

Epididymal fat pads from mice fed LFD vs. VHFD were removed and snap-frozen immediately in liquid nitrogen for subsequent RNA extraction with TRIzol reagent (Invitrogen, Carlsbad, CA). Reverse transcription was done using M-MLV reverse transcriptase and random hexamer primers (Invitrogen). Real-time quantitative PCR was performed with ABI Prism 7500 Sequence Detection System according to manufacture's recommendations (Applied Biosystems), with GAPDH used as an internal control for normalization and the following oligonucleotide primers for each target gene:

ACL, 5′-TGGATGCCACAGCTGACTAC-3′ and 5′-GGTTCAGCAAGGTCAGCTTC-3′;

FAS, 5′AAGTTGCCCGAGTCAGAGAA-3′ and 5′-CGTCGAACTTGGAGAGATCC-3′;

### Statistical analysis

Data are presented as mean±SEM. Differences were analyzed by unpaired two-tailed *t*-test between two groups or otherwise by one-way ANOVA.

## Supporting Information

Table S1 Proteins with altered expression in the WAT of the HFD-fed mice at 16 weeks Numbers with “-” indicate fold decreases in the VHFD-fed group, or fold increases otherwise. Quantification was done with the ImageMaster 2D Elite Software (Amersham Biosciences).
(0.05 MB DOC)
