# Supplementary material for: Leptin Contributes to the Adaptive Responses of Mice to High-Fat Diet Intake through Suppressing the Lipogenic Pathway
Source: PLoS One. 2009 Sep 3;4(9):e6884. doi: 10.1371/journal.pone.0006884 (PMC2731220; doi:10.1371/journal.pone.0006884)
Supplement: Table S1 — Proteins with altered expression in the WAT of the HFD-fed mice at 16 weeks Numbers with “-” indicate fold decreases in the VHFD-fed group, or fold increases otherwise. Quantification was done with the ImageMaster 2D Elite Software (Amersham Biosciences). (0.05 MB DOC) [file pone.0006884.s001.doc]

Supporting Information

Table S1. Proteins with altered expression in the WAT of the HFD-fed mice at 16 weeks

| Accession no. | Gene name | Description | Fold |
| --- | --- | --- | --- |
| NP_598798 | ATP citrate lyase | lipid, fatty acid and isoprenoid metabolism | -21.60 |
| NP_032014 | Fatty acid synthase | lipid, fatty acid and isoprenoid metabolism | -16.56 |
| NP_033414 | Transketolase | pentose-phosphate pathway | -6.40 |
| NP_032641 | NADP-dependent malic enzyme | lipid, fatty acid and isoprenoid metabolism | -5.49 |
| NP_032823 | Pyruvate carboxylase | lipid, fatty acid and isoprenoid metabolism | -3.00 |
| AAH94462 | Aconitate hydratase, mitochondrial precursor | C-compound and carbohydrate metabolism | -2.41 |
| NP_035658 | Transaldolase | pentose-phosphate pathway | -1.99 |
| NP_031462 | Aldehyde dehydrogenase, dimeric NADP-preferring | C-compound and carbohydrate metabolism | -1.87 |
| NP_034401 | Glycerol-3-phosphate dehydrogenase [NAD+] | C-compound and carbohydrate metabolism | -1.72 |
| NP_036167 | Synaptic vesicle membrane protein VAT-1 homolog | intracellular transport vesicles | 1.53 |
| NP_058052 | Enoyl coenzyme A hydratase 1, peroxisomal | lipid, fatty acid and isoprenoid metabolism | 1.63 |
| NP_766594 | Paraoxonase 3 | Stress response,detoxification | 1.71 |
| CAJ18120 | Annexin A1 | signal transduction | 1.76 |
| NP_075608 | Alpha-enolase | C-compound and carbohydrate metabolism | 1.83 |
| CAA31455 | Gamma actin-like protein | cytoskeleton/structural proteins | 1.83 |
| NP_033280 | Serpin B6 | protease inhibitor | 1.90 |
| CAA39807 | Vimentin | cytoskeleton/structural proteins | 1.91 |
| NP_033803 | Annexin A5 | signal transduction | 1.92 |
| NP_031978 | Protein disulfide isomerase associated 3 | signal transduction | 1.98 |
| NP_666232 | Gelsolin | cytoskeleton/structural proteins | 2.21 |
| NP_033934 | Catalase | stress response,detoxification | 2.40 |
| NP_032905 | Plastin-2 | cytoskeleton/structural proteins | 3.01 |

Numbers with "-" indicate fold decreases in the VHFD-fed group, or fold increases otherwise. Quantification was done with the ImageMaster™ 2D Elite Software (Amersham Biosciences).
